# Chromosome-scale assembly uncovers genomic compartmentation of *Fusarium oxysporum* f. sp. *albedinis*, the causal agent of Bayoud disease in date palm

**DOI:** 10.3389/fmicb.2023.1268051

**Published:** 2023-10-11

**Authors:** Slimane Khayi, Andrew D. Armitage, Fatima Gaboun, Issam Meftah-kadmiri, Rachid Lahlali, Mohamed Fokar, Rachid Mentag

**Affiliations:** ^1^Biotechnology Research Unit, Regional Center of Agricultural Research of Rabat, National Institute of Agricultural Research, Rabat, Morocco; ^2^Natural Resources Institute, University of Greenwich, Chatham Maritime, Greenwich, United Kingdom; ^3^Plant and Microbial Biotechnology Center, Moroccan Foundation of Advanced Science Innovation and Research MAScIR, Ben Guerir, Morocco; ^4^Plant and Soil Microbiome Sub-Program, AgroBioSciences, Mohammed VI Polytechnic University (UM6P), Ben Guerir, Morocco; ^5^Phytopathology Unit, Department of Plant Protection, Ecole Nationale D’Agriculture de Meknes, Meknès, Morocco; ^6^Plant Pathology Laboratory, AgroBioSciences, College of Sustainable Agriculture and Environmental Sciences, Mohammed VI Polytechnic University, Ben Guerir, Morocco; ^7^Center for Biotechnology and Genomics, Texas Tech University, Lubbock, TX, United States

**Keywords:** effector, genome, phylogenomics, *Phoenix dactylifera*, pandemic, accessory chromosome, lineage specific

## Abstract

Date palm (*Phoenixdactylifera*) is the most significant crop across North Africa and the Middle East. However, the crop faces a severe threat from Bayoud disease caused by the fungal pathogen *Fusarium oxysporum* f. sp. *albedinis* (FOA). FOA is a soil-borne fungus that infects the roots and vascular system of date palms, leading to widespread destruction of date palm plantations in North Africa over the last century. This is considered the most devastating pathogen of oasis agriculture in North Africa and responsible for loss of 13 million trees in Algeria and Morocco alone. In this study, we present a chromosome-scale high-quality genome assembly of the virulent isolate Foa 44, which provides valuable insights into understanding the genetic basis of Bayoud disease. The genome assembly consists of 11 chromosomes and 40 unplaced contigs, totalling 65,971,825 base pairs in size. It exhibits a GC ratio of 47.77% and a TE (transposable element) content of 17.30%. Through prediction and annotation, we identified 20,416 protein-coding genes. By combining gene and repeat densities analysis with alignment to *Fusarium oxysporum* f. sp. *lycopersici* (FOL) 4287 isolate genome sequence, we determined the core and lineage-specific compartments in Foa 44, shedding light on the genome structure of this pathogen. Furthermore, a phylogenomic analysis based on the 3,292 BUSCOs core genome revealed a distinct clade of FOA isolates within the *Fusarium oxysporum* species complex (FOSC). Notably, the genealogies of the five identified Secreted In Xylem (SIX) genes (1, 6, 9, 11 and 14) in FOA displayed a polyphyletic pattern, suggesting a horizontal inheritance of these effectors. These findings provide a valuable genomics toolbox for further research aimed at combatting the serious biotic constraints posed by FOA to date palm. This will pave the way for a deeper understanding of Bayoud disease and facilitate the development of effective diagnostic tools and control measures.

## 1. Introduction

Date palm (*Phoenix dactylifera*) plays a crucial role in the agrarian economy. It is a key component of the date-palm-based production system known as “oasis agriculture.” This tree is extensively cultivated for its fruits across vast arid regions, spanning from the Atlantic shores to Southern Asia. Recent studies have identified two distinct regions where date palm cultivation originated independently, namely North Africa and the Middle East (Gros-Balthazard et al., 2018). These regions are the primary centres of date palm cultivation worldwide. However, the crop faces significant challenges from a wide range of insect pests and pathogens, resulting in major economic losses (Fernandez et al., 1997; Sedra, 2003; Bouhlali et al., 2020). The western genetic pool in North Africa is particularly vulnerable to the presence of Bayoud disease, a devastating vascular wilt. Morocco, Algeria, and Mauritania have witnessed the loss of millions of date palm trees due to this disease during the last century (Sedra, 2003, 2007, 2012).

*Fusarium oxysporum* f. sp. *albedinis* (FOA) is the causal agent of Bayoud disease, also known as date-palm dieback. This soil-borne fungus is incontestably the most serious fungal disease of date palm. FOA spores and mycelium are the most important means of its transmission. The wilt occurs as a result of root colonization followed by the host vascular system leading to the death of date palms. According to several studies (Louvet and Toutain, 1981; Fernandez et al., 1997, 1998), Bayoud disease has likely originated in Morocco from which it spread to Algeria and Mauritania and now represents an epiphytotic disease which is difficult to control. Moreover, FOA has high incidence across high-quality date palm varieties (e.g., Majhoul, Boufeggous, Deglat Nour) as they are highly susceptible to the infection. For all these considerations, FOA is currently a quarantine pathogen in the European Union and is included in List A2 of the European and Mediterranean Plant Protection Organization.

Recent advancements in sequencing technology have revolutionized our understanding of fungal genome organization and the identification of genome-scale variations. Several genomes of *Fusarium oxysporum* (FO) fomae speciales (ff. spp.) have been sequenced and analysed, with *Fusarium oxysporum* f. sp. *lycopersici* (FOL) serving as the reference genome and being the most extensively studied taxon (Ma et al., 2010). Comparative analysis of FO genome sequences has revealed highly dynamic structure characterized by a compartmentalization into core (CC) and lineage-specific chromosomes (LS). The core chromosomes primarily encode the house-keeping functions of the cells and core effectors, while the lineage-specific chromosomes encode plant host-specific virulence genes (Ma et al., 2010; Armitage et al., 2018). These lineage-specific regions, often referred to as accessory chromosomes, lack synteny with other *Fusarium* species and are characterized by a relatively high number of repeats and lower gene density (Ma et al., 2010, 2013; Galazka and Freitag, 2014).

Until 2020, no genome sequence was available for FOA. Subsequently, two genome sequences of FOA were sequenced and published in 2020 by Khayi et al. (2020) and in 2022 by Khoulassa et al. (2022). While several studies have since been published on FOA, none have yet characterized the genome structure of this important pathogen (Ayada et al., 2022; Rafiqi et al., 2022). Hence, a significant knowledge gap persists regarding the arrangement of the FOA genome and the genetic mechanisms driving its pathogenic properties. To address this gap, we present a chromosome-scale high-quality genome assembly of FOA and a detailed analysis of its genome structure, focusing on CC and LS chromosomes and their potential role in determining host preference. Our work provides important new insights into the genomic basis of FOA genome organisation. We believe that, understanding the genome structure of FOA holds immense potential for the development of effective disease control strategies such as developing efficient tools for diagnosis based on the LS regions that could harbor specific ff. spp. genes and/or highly diverse DNA sequences suitable for marker development. The establishment of effective diagnostics will facilitate enhanced disease management, thereby yielding better control over this threat to oasis agriculture.

## 2. Materials and methods

### 2.1. Foa 44 cultivation and DNA extraction

The FOA Foa 44 isolate (=CCMM/INRA Foa/44, =BCCM MUCL 41814) was recovered from the date palm cv. Majhoul in the Tafilalt-Rissani region of Morocco in 1981. The isolate was cultured on potato dextrose agar medium (PDA) plate in 25°C for 7 days. The mycelia collected from single spore cultures were used for DNA extraction using CTAB method (Möller et al., 1992). 50 mg of lyophilized mycelia was mixed with 1 ml of CTAB extraction buffer (1 M Tris–HCl, 5 M NaCl, 0.5 M EDTA, 2% (w/v) CTAB and 0.02% (v/v) of β-mercaptoethanol) and incubated to 65°C for 1 h. Following this, chloroform isoamyl alcohol (24:1-v/v) was added and the mixture was centrifuged at 13,000 rpm for 10 min. The supernatant was recovered in a new tube and precipitated overnight by isopropanol. Finally, the DNA was washed with 200 μl of ethanol (75%) and dissolved with sterile ultra-pure water. The quality and the quantity of DNA extraction was assessed using agarose gel electrophoresis at 1.0%, a NanoDrop ND1000 device, and Qubit 2.0 fluorometer.

### 2.2. Genome sequencing and assembly

Sequencing of Foa 44 genomic DNA was performed using Illumina short read technology, and also generated long-read data for this strain using Nanopore technology. To prepare for Illumina sequencing, we used the Nextera DNA Flex library kit to construct a paired-end library with 0.5 μg of total genomic DNA. The library was sequenced on a NovaSeq 6000 platform (2 × 150 bp). In addition to this, whole-genome sequencing was also performed on the Oxford Nanopore Technologies MinION platform, using the rapid barcoding kit (SQK-RBK004). The library was prepared according to the manufacturer protocol starting with 400 ng of total genomic DNA. The clean-up step was performed using Agencourt AMPure XP beads to remove short fragments andsequencing of the prepared library carried out using a MinION Mk1b sequencer (MinION flow cell R9.4.1), and raw sequencing data were base called and analysed using the Guppy base caller v5.1.13 (Wick et al., 2019) and MinKNOW v4.5.4.

A hybrid approach was used for genome assembly with the MaSuRCA assembler (v3.4.1; Zimin et al., 2013) which took both the Illumina and Nanopore reads as input. We used specific parameters in the assembly process (JF_SIZE = 9,000,000,000; CA_PARAMETERS = cgwErrorRate = 0.15; SOAP_ASSEMBLY = 0; FLYE_ASSEMBLY = 0). Following this, BUSCO was used to evaluate the completeness of the genome assembly using the sordariomycete lineage-specific profile library (sordariomyceta_odb10).

### 2.3. Identification of repeat elements and annotation of the genome sequence of Foa 44

The repeat sequences in the Foa 44 genome sequence were identified and masked before processing its annotation. RepeatModeler (Flynn et al., 2020) was used to build a *de novo* repeat library based on the Foa 44 assembly, and then RepeatMasker (Tarailo-Graovac and Chen, 2009) was applied against Dfam V3.151 using specific parameters “-nolow, -no_is, -norna, -parallel 56.” The Fungap pipeline was used for the prediction and annotation of Foa 44 gene models (Min et al., 2017). The pipeline Funannotate (Palmer and Stajich, 2017) was used for functional annotation of the predicted genes against six databases: Pfam, CAZy, MEROPS, eggNOG, InterProScan (v5.20–59.0), and UniProt. Additionally, SignalP (version 5.0b) was utilized to identity the secreted proteins among predicted genes.

### 2.4. Identification of secreted in xylem profile of Foa 44

To identify homologs of the 14 known SIX (Secreted In Xylem) genes from FOL, searches within the genome assemblies of FOA isolates we performed using the command line tool *blastn*, and *tblastn*. Initially, Blast databases were established using both the entire genome sequences and, when accessible, the coding sequence (cds) transcripts of FOA isolates. This was achieved utilizing the command ‘makeblastdb -in in.cds-transcripts.fa -dbtype nucl -out db/out’. Subsequently, the inquiry was executed through the script ‘tblastn/blastn -query input.fa -db db -evalue 0.1 -out results.out -outfmt “6 qseqid sseqid pident length mismatch gapopen qstart qend sstart send evalue bitscore qlen”.

### 2.5. Foa 44 genome alignment and identification of lineage specific regions

To identify LS and CC regions in Foa 44, we applied a reference-guided assembly approach. First, the whole assembly was scaffolded into pseudomolecule chromosomal scaffolds using RaGOO (Alonge et al., 2019), with the FOL Fol 4287 genome assembly serving as the reference.

Ragoo scaffolding relies on Minimap2 (Li, 2018) alignment of the initial contigs to a reference genome, producing a chromosome-level assembly of the query that indicates the corresponding chromosomes in the reference sequence. Any unclassified sequences were considered putative LS contigs. To assess the consistency of the reference-guided scaffolding, we investigated the collinearity of Foa 44 to Fol 4287 using MACSanX tool (Wang et al., 2012). The coverage analysis was performed by mapping of Foa 44 trimmed reads on Fol 4287 genome sequence using BWA-MEM and the genome coverage was calculated using BamToCov tool (Birolo and Telatin, 2022). The plot was generated using KaryoploteR package in R (Gel and Serra, 2017).

### 2.6. Phylogenetic relationship of FOA isolates within *Fusarium oxysporum* complex

We conducted a phylogenomic analysis of the Foa 44 including the available 3 isolates strain9, Foa 133 and 13116 using BuscoPhylo web server (https://buscophylo.inra.org.ma/; Sahbou et al., 2022). The analysis was performed with 34 genome sequences of ff. spp. isolates as input, and *F. graminearum* PH-1 and *F. culmorum* UK99 was used as an outgroup (Supplementary Table S1). The workflows and scripts used to perform analysis and generate plots are deposited in the following GitHub repository: https://github.com/SolayMane/FOA_scripts_final.

## 3. Results

### 3.1. Whole genome sequence assembly of Foa 44

Genome sequencing of the Foa 44 isolate yielded a total of 212,836,043 Illumina reads and 497,587 Nanopore reads with an average length of 147 and 3,111 bp, respectively. The N50 and N90 of Nanopore reads are, respectively, 7,806 and 1,717 bases. Analysis of the 21-mer frequency distribution within the 520X coverage of trimmed Illumina data allowed for an estimation of the Foa 44 genome size, which was calculated to be 57,144,323 bp with single-mode curve profile confirming the haploid character of FOA genome (Supplementary Figure S1). Based upon the generated data, the hybrid *de novo* assembly with MaSuRCA, produced a genome sequence consisting of 68 contigs totalling 65,970,125 bp in size with a GC ratio of 47.75%. The N50 of the contigs-based assembly reached 2,783,938 bp, and the largest contig was 6,425,555 bp in length (Table 1).

**Table 1:** Statistics of Foa 44 genome assembly.

	Initial assembly	Scaffolded assembly
Number of Contigs	68	51
Largest contig (bp)	6,425,555	6,425,555
Total length (bp)	65,970,125	65,971,825
GC (%)	47.8	47.8
N50 (bp)	2,783,938	4,061,178

Reference-guided scaffolding of the Foa 44 genome, based on the Fol 4287 genome sequence, allowed the assembly of 28 Foa 44 contigs into 11 pseudochromosomes (Chr1, Chr2, Chr4, Chr5, Chr7, Chr8, Chr9, Chr10, Chr11, Chr12, and Chr13) displaying high grouping scores to 11 CCs of Fol 4287 (Supplementary Table S2). The remaining 40 contigs that did not align against the reference genome were considered as Foa 44 LS contigs. The N50 of the Foa 44 scaffolds was 4.061 Mb, with the maximum scaffolds size was 6.4 Mbp, and the smallest contigs reached 11,931 bp (Table 1). CCs of Foa 44 represented 45.07 Mbp of the Foa 44 assembly while the remaining LS contigs totalled 20.9 Mbp of the assembly.

The assessment of Foa 44 genome completeness was performed using 3817 BUSCO Sordariomycete fungal genes. The results showed that Foa 44 harboured 98% (3,470) of the genes in a complete orthologs, 97% (3,702) Complete and single-copy BUSCOs, 1% (38) Complete and a lower fraction of missing 1.4% (56) and fragmented 0.6% (21) genes, respectively. These results indicate a high-quality genome assembly for the Foa 44 genome sequence.

**Figure 1:**
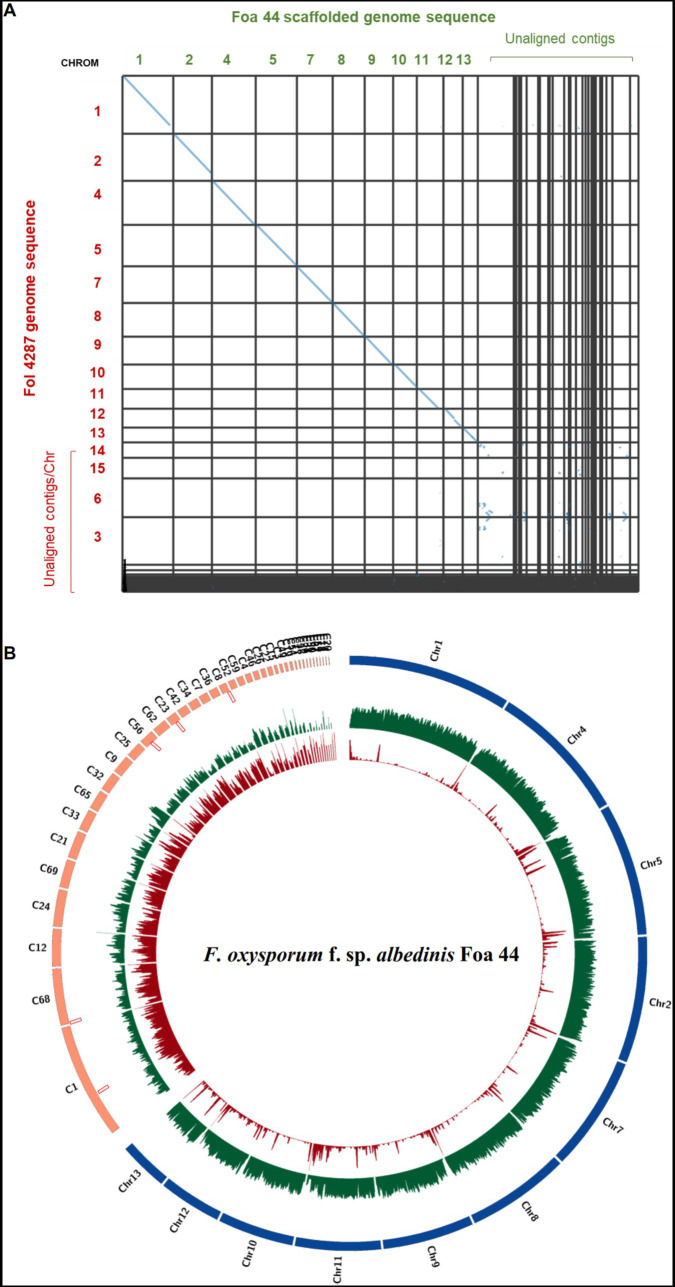
**(A)** Dot plot showing homologous chromosomes between Fol 4287 and Foa 44. The dot plot was generated using SynVisio tool based on MCScanX collinearity gene output. Every collinear gene is represented by a blue point and contiguous collinear blocks are shown as lines. **(B)** Circos plot of FOA Foa 44 genome sequences. Chromosomes aligned to Fol 4287 are coloured in blue and the unaligned Contigs are coloured in orange. Tracks from outer to inner part indicate TEs densities, Gene densities, and SIX genes location, respectively.

The analysis using MCScanX tool have indicated that Fol 4287 and Foa 44 share 69.96% of collinear genes (29,215 of 41,761). The synteny dot plots generated by MCScanX have demonstrated nearly complete alignment of the 11 CC between Foa 44 and Fol 4287, while the LS chromosomes are poorly aligned (Figure 1A).

### 3.2. Gene prediction and annotation of Foa 44 strain

**Figure 2:**
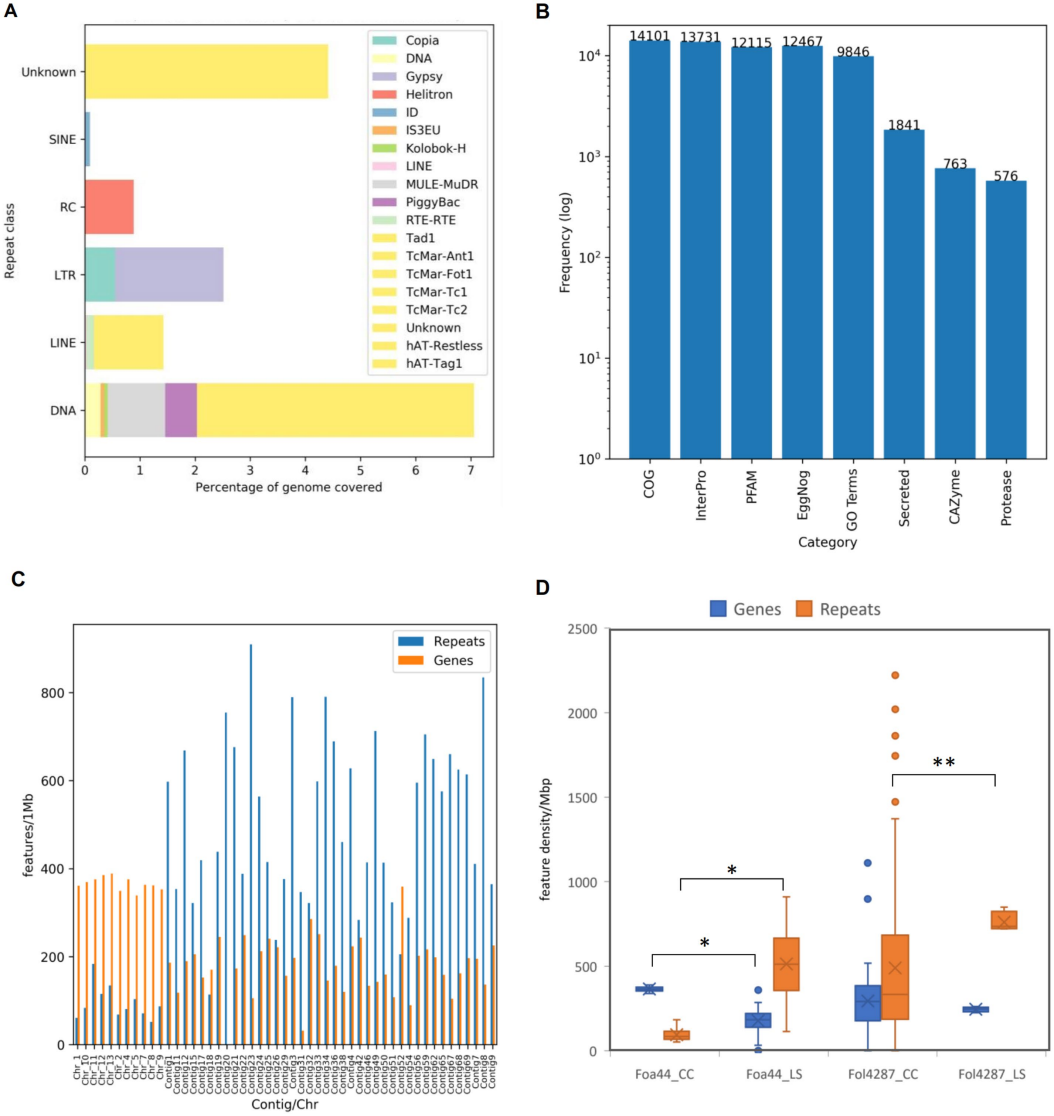
Statistics of TEs and genes prediction and annotations of Foa 44 genome sequence. **(A)** Frequencies of identified TE by class and family. **(B)** Frequencies of features annotations based on homologies against specific databases. **(C)** TEs and Genes densities across LS and CC regions in features per 1Mbp. **(D)** Distribution of Genes and Repeats densities across CC and LS regions for Foa44 and Fol 4287. Significant difference of means of genes and repeats between CC and LS as calculated using ANOVA test shown by bars (*p < 0.0001, **p < 0.0003).

Prior to gene prediction, Transposable Elements (TEs) were identified and masked in the Foa 44 genome. A total of 17.30% (11,411,057 bp) of the full length was masked, encompassing 0.10% (31) of Short Interspersed Nuclear Elements (SINEs), 1.43%% (860) of Long Interspersed Nuclear Elements (LINEs), 2.51% (1,061) of Long Terminal Repeats elements (LTRs), 7.07% (4,445) of DNA transposons elements and 4.42% (8,078) of Unclassified portion of repeats sequences and 0.89% (365) of the repeats were classified as rolling-circles repeats (Figure 2A). The analysis of TE content highlights the prevalence of DNA transposons as the most abundant type of TEs in the Foa 44 genome sequence, consistent with the findings in other FO isolates (Schmidt et al., 2013; van Dam et al., 2017; Armitage et al., 2018). Interestingly, FOA isolates showed a higher TEs content compared to other FO isolates such Fol 4287 (14.34%) and Fus2 (10.14%) (Supplementary Table S3) which may reflect rapid genome evolution as a result of TE activity within FOA. The gene annotation of Foa 44 resulted in the identification of 20,416 protein-coding genes, including 14,086 (86.99%) spliced genes. The average gene density was found to be 351 genes per 1 Mbp across the entire genome sequence of Foa 44. To functionally annotate the genes, we utilized six databases: Pfam, CAZy, MEROPS, eggNOG, InterProScan (v5.20–59.0), and UniProt. The results of these annotations are presented in Supplementary Table S4. Overall, we were able to annotate more than 89.9% (18,171 genes) of the protein-coding genes with at least one of the six databases. Specifically, we found that 12,115 (59.34%), 763 (3.73%), 13,731 (67.25%), 12,467 (61.06%), 9,846 (48.22%), and 14,101 (69.06%) protein-coding genes were matched with the PFAM, CAZyme, InterPro, EggNog, GO, and COG databases, respectively (Figure 2B). The gene count predicted were comparable to those of various others isolates of *F. oxysporum* such as Fol 4287 (21,354) and N139 (20,493; Supplementary Table S3).

Carbohydrate-active enzyme (CAZyme) are classified into six class of enzymatic activities based amino-acid sequence and structure similarities. They play an important role in the degradation, modification and biosynthesis of carbohydrates (Cantarel et al., 2009). CAZymes annotations showed that the Foa 44 strain genome sequence is enriched with CAZymes accounting a total of 801 families assigned to 763 genes representing 3.72% of the total number of genes. Notably, our analysis revealed a higher number of CAZymes in Foa 44 compared to previous studies conducted on Foa 133 (Ayada et al., 2022; Rafiqi et al., 2022). The highest number of CAZyme genes (374) was assigned to Glucoside Hydrolase (GH) family, followed by Carbohydrate Esterases (CE; 128), Auxilary Activities (AA; 119), Glycosyl Transferases (GTs; 103), Polysaccharide Lyases (PL; 27), and Carbohydrate-Binding Modules (CBMs; 12). When compared to other isolates of *Fusarium oxsporum* ff. spp., the Foa 44 genome was found to be among those richest in CAZymes within FO isolates in a range of 949 for *F. oxysporum* f. sp. *melonis* 26,406 and and 688 for f. sp. cubense TR4.

Among the predicted protein of Foa 44, 9.1% were found to contain N-terminal peptides (SignalP v4) accounting for 1,841 genes, compared to 1,464 genes for Foa 133 (Rafiqi et al., 2022), after filtering out proteins adhered to membranes or targeted to other subcellular compartments. The differences observed in the secreted proteins content between Foa 44 and Foa 133 may be attributed to the quality of the assemblies and the methods employed for genome sequences mining. Similar percentages were observed across isolates from other ff. spp. Additionally, scanning the Merops database highlighted the presence of 561 genes that are suggested to be proteases (Figure 2B). The FOA isolates carried fewer proteases compared to Fol 4287 and Fom 26406 possessing 770 and 758 genes, respectively (Supplementary Table S3).

### 3.3. Gene and repeat densities allow core and accessory chromosome identification in Foa 44

LS chromosomes are typically characterized by low gene density and high repeat density, whereas CCs are gene-rich and have lower repeat densities (Ma et al., 2010). In this study, the partitioning of the Foa 44 genome sequence into Core and LS chromosomes/Contigs was substantiated through examination of gene and TEs densities. TEs distribution analysis across the genome divulged that approximately 73.7% of repeats were located in LS contigs, exhibiting an average density of 513 TEs per megabase (TEs/Mbp). Conversely, the remaining 27% were localized within 11 CCs, with an average density of 94 TEs/Mbp (Figure 1B, 2C; Supplementary Table S5). Upon scrutinizing gene densities, CC exhibited notably higher average density (365 genes/Mbp) in comparison to LS contigs (177 genes/Mbp; Figures 1B, 2C; Supplementary Table S5).

To strengthen the delineation of Foa 44 genome compartmentation, a comprehensive visualisation of the synteny between Foa 44 and Fol 4287 was constructed, capitalizing on collinearity data in conjunction with gene and repeat densities (Supplementary Figure S2). The findings underscored a near-syntenic relationship between the 11 CCs of Fol 4287 and those of Foa 44, as depicted in the dot plot (Figure 1A). This genomic structure was further underpinned by variations in TE and gene densities across CC and LS contigs. Moreover, a coverage analysis was conducted by mapping the trimmed Foa 44 paired-end reads onto Fol 4287, revealing low coverage on Fol 4287 LS Chromosomes 3, 6, 14, and 15, which supports our findings (Supplementary Figure S3). Significant differences in gene and TEs densities were observed between the CC and LS contigs within the Foa 44 genome. However, in the case of the Fol 4287 genome, a significant difference was only noted in the distribution of TEs between CC and LS (Figure 2D; ANOVA, *p*-value <0.0001).

**Figure 3:**
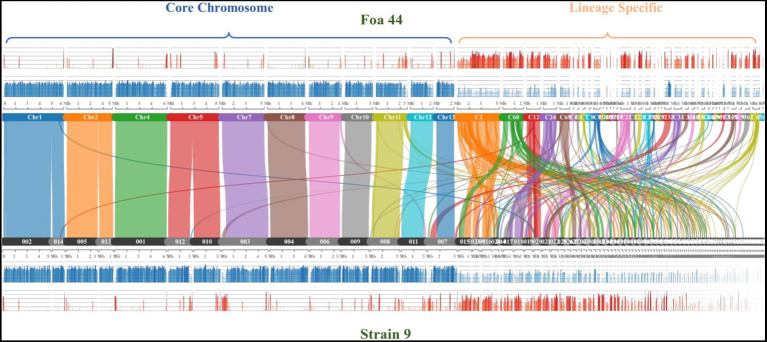
Comparison of syntenic orthologue blocks (2) between the Foa 44 chromosomes/contigs and strain 9 Scaffolds. The histograms represent the Gene (blue) and TEs (red) densities across the genome sequences.

To further confirm the genome compartmentation of the FOA genome, we performed collinearity analysis between the two FOA genome sequences Foa 44 and strain 9, using the MCScanX tool (Figure 3). The results showed that the two genome sequences shared 81.47% (32,689 out of 40,125 genes) of collinear genes, and the dot plot highlighted the synteny between the 11 pseudo-core chromosomes of Foa 44 and the strain 9 scaffolds 2, 14, 5, 13, 1, 12, 10, 3, 4, 6, 9, 8, 11 and 9. Considering gene and repeat densities, the plots clearly showed the same pattern of fluctuations between the LS and CC in both genomes. Overall, this analysis highlights for the first time the genome compartmentation across FOA genomes, which offers an important genomic toolbox for further research on this pathogen.

### 3.4. Phylogenomics confirms a single clade of date palm-infecting *Fusarium oxysoporum*

**Figure 4:**
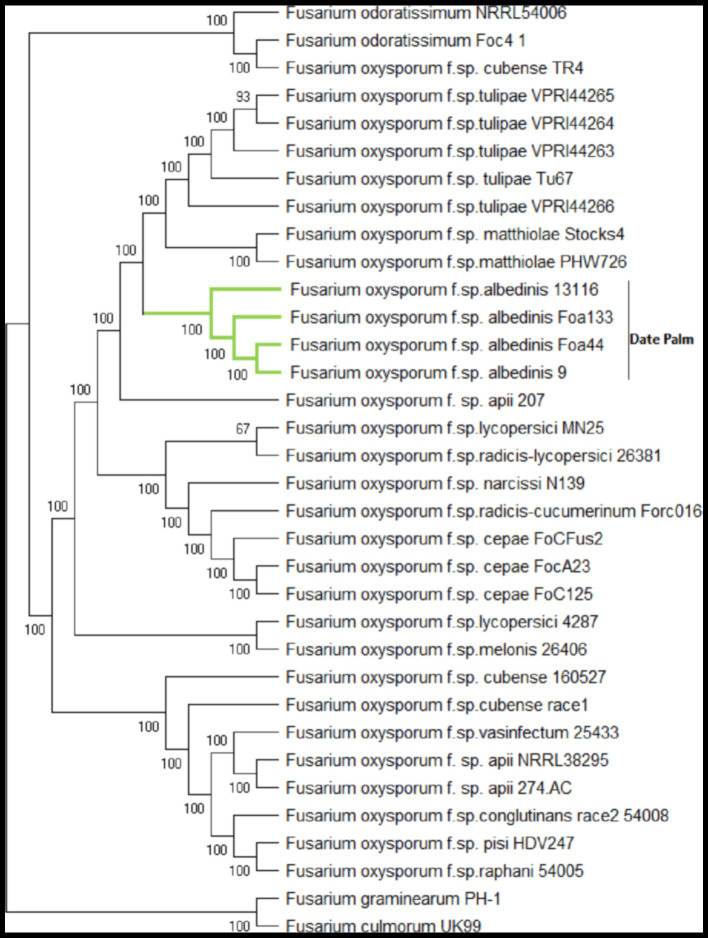
Maximum-likelihood phylogenomic tree generated based on shared 3292 BUSCOs protein sequences across 34 genome sequences. The amino acid sequence of the translated genes was concatenated, and the final alignment consists of a total of 1,918,665 amino acids. The phylogenetic tree was constructed using BuscoPhylo pipeline, *F. graminearum* PH-1 and *F. culmonorum* UK99 were used as outgroup.

To determine the taxonomic position of FOA isolates within the FO complex species (FOSC), we constructed a phylogenetic tree using BUSCO single-copy genes shared among 34 Fusarium genomes. A total of 3,292 BUSCOs were identified as single copy in all 34 genomes. The individual alignments were trimmed and concatenated, resulting in a supermatrix alignment with a length of 1,918,665 amino acids. The phylogenetic tree exhibited a similar topology to previous studies, demonstrating the polyphyletic nature of FO species and ff. spp. that infect specific host plants (van Dam et al., 2016; Armitage et al., 2018; Batson et al., 2021; Jenkins et al., 2021; Figure 4).

Both strains of *F. odoratissimum* (Foc4 and NRRL54006) and *F. oxysporum* f. sp. *cubense* TR4 formed separate clades, consistent with their designation as distinct *Fusarium* species (Lombard et al., 2019; Maryani et al., 2019). FOA isolates were grouped in a unique sub-clade with a high bootstrap value of 100%, indicating that they constitute a distinct *formae speciales* to date palm. Specifically, the FOA sub-clade showed close position with *F. oxysporum* f. sp. *mathiolae* and f. sp. *tulipae*. This taxonomic analysis represents the most comprehensive phylogenomic tree to date, providing insights into the phylogenetic relationships of FOA with other *F. oxysporum* isolates.

Notably, unlike several polyphyletic *formae speciales* such as tomato and cucurbit strains, these FOA isolates, originating from different host cultivars and geographically distant locations, were observed to form a monophyletic *forma specialis*. These findings support the hypothesis of FOA’s clonality as a pathogen, suggesting a common ancestry.

Overall, the phylogenetic analysis based on the comprehensive set of BUSCO genes provides valuable insights into the taxonomic position of FOA within the FOSC. These findings contribute to our understanding of FOA’s evolutionary history and shed light on its unique genetic characteristics within the complex.

### 3.5. FOA genome analysis reveals a limited set of SIX genes

The SIX genes play a crucial role in the pathogenicity of FO. These genes encode effector proteins that help the fungus colonize plant tissues by suppressing the host immune response (Houterman et al., 2007). In this study, we conducted a *blastn* and *tblastn* search to identify homologs of the 14 known SIX genes in FOL across all available FOA isolates.

**Figure 5:**
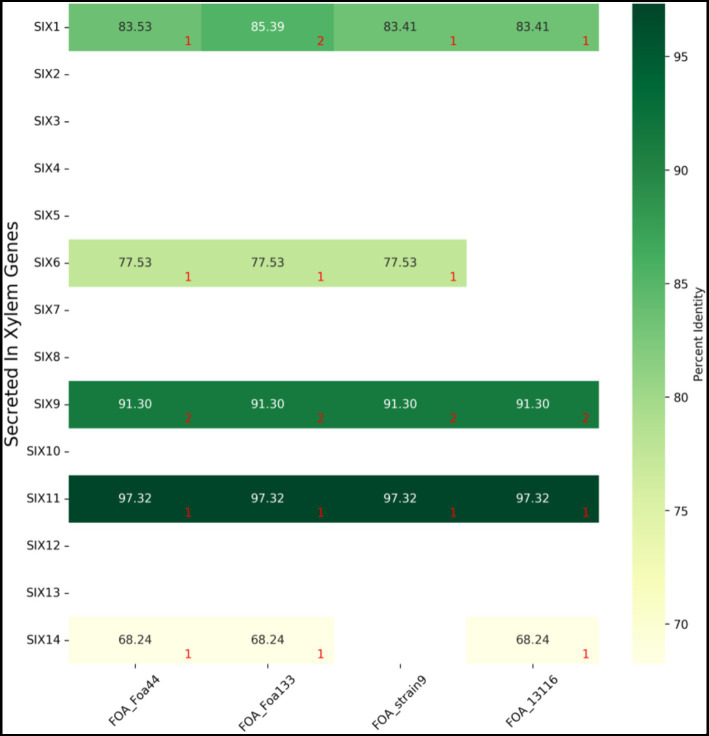
BLASTn results of 14 *F. oxysporum* f.sp. *lycopersici* SIX gene sequences against four FOA genome sequences. Numbers in red indicate the number of copies found by blastn. Detailed results are summarized in Supplementary Table S6.

Our *blastn* and *tblastn* results show that only five SIX genes (1, 6, 9, 11, and 14) were present in FOA genomes, while the remaining nine (2, 3, 4, 5, 7, 8, 10, 12, and 13) were absent (Figure 5; Supplementary Table S6). All the five SIX genes were present in FOA isolates, except for strain 9, which lacked the genes SIX14 and strain 13116, which lacked the SIX6 gene. Additionally, two copies of the SIX9 gene were found in all FOA isolates, and the isolate Foa133 harboured a fragmented SIX1 gene that spanned across two contigs. Notably, all the SIX genes were located in the designated LS regions in Foa 44 and strain 9 isolates.

To confirm that the SIX genes were not misassembled in the Foa 44 genome, we mapped the trimmed reads of Foa 44 against the 14 SIX genes. We obtained a consensus read mapping only for the genes that were found by the *blastn* and *tblastn* search. This limited set of SIX genes in FOA isolates suggests that this pathogen may have evolved unique mechanisms to cause disease in date palm plants. Further functional investigations are required to gain a better understanding of these mechanisms.

### 3.6. Phylogenetic analysis of SIX genes in FOA isolates

Phylogenetic analyses were conducted on each of the five SIX genes present in FOA within the FOSC to highlight their evolutionary relationships. Homologs of these five genes were screened against 229 genome sequences of different ff. spp. The present SIX genes were aligned to infer the individual phylogenetic trees.

**Figure 6:**
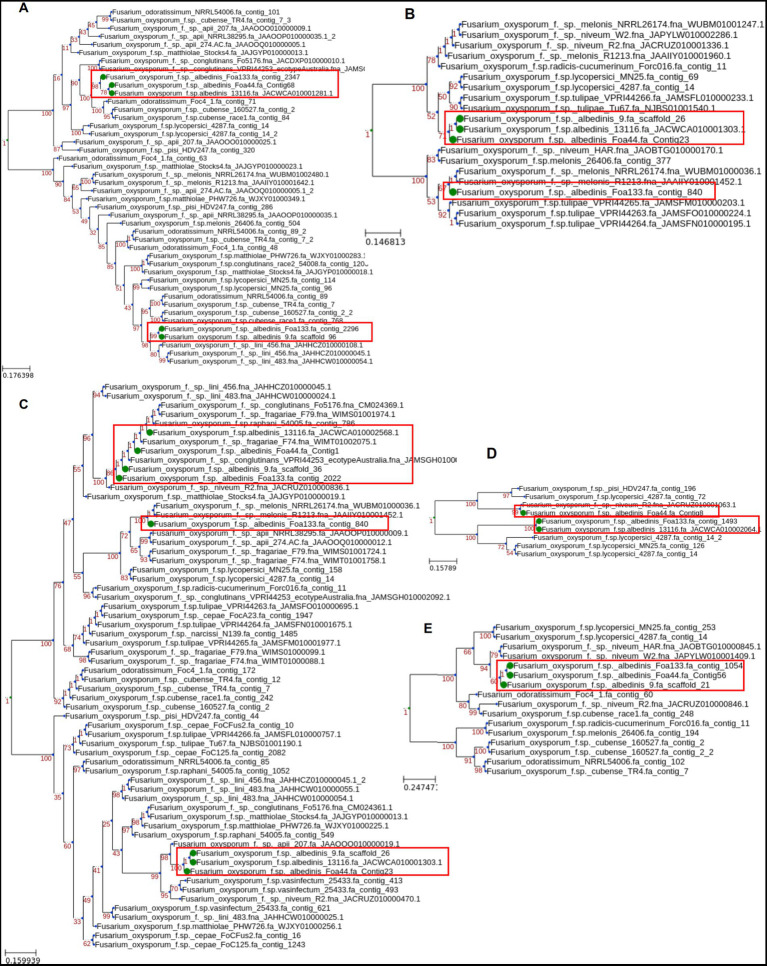
Maximum-likelihood phylogenetic tree of SIX genes from FOA isolates. The analysis was conducted using homologs of SIX genes 1 **(A)**, 11 **(B)**, 9 **(C)**, 14 **(D)**, and 6**(E)** found in the genome sequence of other ff. spp.

The phylogenetic analysis of SIX gene sequences revealed a variable topology of the tree across the SIX genes (Figure 6). The FOA SIX genes were clustered in several separated clades alongside other *formae speciales*, except for the SIX6 gene where Foa133, Foa 44, and strain 9 share a recent common ancestor of SIX6 with f. sp. *niveum* (watermelon; Figure 6E).

For the SIX1 gene (Figure 6A), the FOA homologs were distributed within two separate clades within FOSC, suggesting multiple sources of the SIX genes acquisition. For the SIX9 gene (Figure 6C), two variants were detected in FOA isolates and clustered in three clades. The first variant (Foa 44_contig1, strain 9 scaffold_36, Foa133_contig2022, and strain 13116_JACWCA010002568.1) is grouped within ff. spp. *fragariae* (strawberry), *raphani* (radish), and *conglutinans* (brassica crops), while the second group is grouped with ff. spp. *apii* (celery) and *vasinfectum* (cotton). Interestingly, for the strain Foa133, SIX9 and SIX11 genes, present in contig_840, were grouped into separate clades with f.sp. *melonis* (melon), suggesting a common source of horizontal gene acquisition (Figures 6C,B).

For the SIX11 gene, we observed two clades of FOA isolates, one containing Foa 44, 9 and13116 and the second harbouring solely Foa133 with ff. spp. *melonis* (Figure 6B). The SIX14 gene was found in Foa 44, 13116, and Foa133 with two groups. The first group harbouring Foa 44 where SIX14 gene was more similar to the homolog in ff. spp. *niveum* while the second group harbouring Foa 133 and 13116 were close to SIX14 detected in ff. spp. *lycopersici* (tomato; Figure 6D).

Based on the overall phylogenetic analysis, the phylogenetic trees of the FOA SIX genes do not show the same topology as conserved in core genes. This opens a hypothesis that FOA SIX genes could have been horizontally transferred from other organisms that coexists in the same agrosystem as date palm.

## 4. Discussion

Over the last century, FOA, the fungus that causes Bayoud disease, has been considered the most devastating pathogen of oases in North Africa and particularly in Morocco (Sedra, 2003). Since its identification, it has been reported that this pathogen has been responsible for the death of approximately 3 and 10 million trees in Algeria and Morocco, respectively (Sedra, 2003; El Modafar, 2010; Bouhlali et al., 2020). Over the past five decades, intensive research activities have been undertaken to reduce the impact of this pathogenic fungus on date palm groves. These efforts have led to the establishment of integrated control approaches based on prophylactic measures, selection and utilization of resistant cultivars, chemical control, and recently the emergence of biological control approaches (Toutain and Louvet, 1974; Sedra, 2007; Bouhlali et al., 2020; Bouissil et al., 2022; Anli et al., 2023). However, all of these aspects have been developed without a comprehensive understanding of the genomic characteristics of the pathogen, which may have limited the efficacy of these approaches. Fortunately, recent advancements in high-throughput sequencing technologies (second and third generation) have paved the way for significant progress. Thanks to the increased accessibility of DNA sequencing using NGS technologies, several genomes have been recently published, ushering in a new era in the fight against FOA (Dobbs et al., 2020; Khayi et al., 2020; Khoulassa et al., 2022).

In this study, we leveraged the advantages of short and long read sequencing technologies to sequence and assemble the genome of Foa 44 isolates at a high-quality chromosome scale, marking a significant milestone. Reference-guided approaches are suitable for organizing assemblies into ordered and contiguous pseudomolecules based on an available reference sequence. However, this procedure can be quite constraining and may introduce reference sequence biases, potentially leading to unreliable genomic information. Therefore, to validate the accuracy of reference guided Foa 44 assembly, independent Foa 44 genome map data such as Hi-C or Bionano, should be generated to ensure the integrity of the assembly.

Compared to the other ff. spp. the genome sizes vary among the isolates, with Foa 44 having one of the largest genome sizes, among *F. oxysporum* ff. spp., of 65.9 Mbp (compared to 65.5 Mbp for strain 9). It’s also, exhibits a higher average scaffold size compared to most isolates, with an exceptionally large largest scaffold size and a notable scaffold N50 of 4.06 Mb (compared to 2.9 Mb for strain 9) and lower number of Scaffolds/Contigs of 51 (compared to 114 for strain 9; Supplementary Table S3). Furthermore, the annotation process for Foa 44 resulted in the generation of 20,416 gene models, amending the 19,411 gene models identified for strain 9.

TEs are known to play a key role in genome diversity in eukaryotes and prokaryotes (Castanera et al., 2016; Alseekh et al., 2020; Gonçalves et al., 2020). With the increased number of sequenced fungal genomes, a recent analyses of variations in TEs content have been linked to the fungal lifestyle rather than phylogenetic affiliation (Castanera et al., 2016). Our examination of the Foa 44 genome sequence revealed a TEs content of 17.3%, while a recent study reported that the strain 9 and Foa 133 showed, respectively, a TEs content of 16.75 and 4.9% of the total genome sequences (Ayada et al., 2022). This intra-*forma specialis* TE variation may be attributed to differences in TE mining methods and the completeness of genome sequences. However, TE density analysis combined with the Fol 4287 whole genome alignment allowed the subdivision of the Foa 44 genome into LS and CC compartment, consistent with previous observations in various FO ff. spp. (Ma et al., 2010; van Dam et al., 2017; Armitage et al., 2018), and other plant-pathogenic filamentous fungi (Coleman et al., 2009; Goodwin et al., 2011; Hu et al., 2012; Armitage et al., 2020). Furthermore, it has been suggested that TE richness of LS chromosomes across ff. spp. is key to underlying lifestyle flexibility in the group (Ma et al., 2013; Hill et al., 2022), a process that may also be key to shaping 20.9 Mb of TE-enriched LS regions in Foa 44.

While a clear conservation of synteny was observed between Foa 44 and Fol 4287 CCs, Fol 4287 LS chromosomes (chromosomes 3, 6, 14 and 15) showed highly reduced synteny with LS contigs of Foa 44 over these entire chromosomes and parts of chromosome 1 and 2, congruent with findings reported by Ma et al. (2010). Based on these designations, we observed a comparable size of LS regions in Foa 44 and Fol 4287 totalling, 20.9 Mbp and 19 Mbp, respectively. In contrast, recent studies revealed an unbalanced size of LS fractions, versus the reference Fol 4287 LS, for Foc and Force, respectively, showing only 5.7 Mb and 2.47 Mb (van Dam et al., 2017; Armitage et al., 2018). It has been shown that LS chromosomes could be transferred or lost during the evolution without affecting the growth of the fungi (Vlaardingerbroek et al., 2016) suggesting that they are not essential for the survival of the pathogen, but potentially for conferring functional advantage (Guo et al., 2021; Hill et al., 2022). This organization of LS regions and CCs has been suggested to allow the fungal population to adapt more quickly to changing conditions imposed by their host (Croll and McDonald, 2012; Raffaele and Kamoun, 2012; Hill et al., 2022).

Previous studies have demonstrated a varied origin within the FOSC, with some ff. spp. showing a polyphyletic origin, while others have a monophyletic origin. This suggests a complex evolutionary history within the FOSC group (Epstein et al., 2017; Armitage et al., 2018; Dobbs et al., 2020; Simbaqueba et al., 2021).

The phylogenomic analysis of the four FOA isolates employing a core genome of 3292 BUSCOs protein sequences, unveiled a single distinct clade within FOSC. These results may indicate a unique origin of FOA on date palm, supporting the hypothesis that the FOA population most likely originated from a single virulent clone that emerged in the Moroccan oases, where the Bayoud disease is prevalent, before spreading to other regions *via* different mechanisms (Tantaoui, 1996; Fernandez et al., 1997). These findings highlight the importance of understanding the evolutionary dynamics of FOA and its implications for managing Bayoud disease.

The 14 known SIX genes discovered in FOL and used to profile other ff. spp. (van Dam et al., 2016; Czislowski et al., 2018; Achari et al., 2021; Batson et al., 2021), were investigated in this study to assess their presence in the four FOA isolates. The results unveiled a narrow set of these effectors, namely homologs of SIX1, SIX6, SIX9, SIX11, and SIX14, extending the repertoire of known SIX genes within the FOA species. Notably, Rafiqi and colleagues have reported only 3 SIX genes (2 variants of SIX1, SIX9 and SIX11). However, our analysis of Foa 133 resulted in the presence of 5 SIX genes: SIX1 gene spanning two contigs (2 variants of SIX1 in Rafiqi et al., 2022), SIX6, SIX9 (2 variants), SIX11 and SIX14. Therefore, our results confirm the presence of SIX1, SIX9, and SIX11 and expand on the previous findings (Ayada et al., 2022; Rafiqi et al., 2022). The homologs of these 14 known SIX genes have been identified in various ff. spp. of *F. oxysporum* that infect a range of plant species, including alliaceous, legumes, musaceous, solanaceous, and narcissus. (Williams et al., 2016; Armitage et al., 2018; Czislowski et al., 2018; Simbaqueba et al., 2021).

To gain further insights into the evolutionary history of the identified SIX genes in FOA genomes, we conducted individual phylogenetic analyses in comparison to FOSC isolates. The phylogenetic analyses revealed a polyphyletic pattern for the SIX genes (1, 9, 11, and 14), where FOA isolates homologs were sub-grouped into different clades within the phylogenetic tree. Notably, the SIX6 sequences for FOA isolates clustered together with other *formae speciales*, which was broadly congruent with the core genome phylogeny (Figure 4). Furthermore, we explored the range of plant hosts associated with these phylogenetic relationships, we observed that for SIX11 a subgroup of FOA isolates (strains 9, 13116, and Foa 44) sharing close homology with isolates infecting tulips, while Foa133 sharing greater homology to isolates infecting melon. Additionally, the SIX genes 9 and 14 exhibited a shared ancestry between FOA isolates and isolates from various host plants, including melon, cruciferous plants, watermelon, and tomato. Overall, the gene tree analysis revealed that the evolutionary history of SIX gene homologs in FOA are incongruent, which would suggest that these determinants of pathogenicity have been inherited both vertically and horizontally within the single core-genome lineage.

In the context of oasis agricultural systems, it is noteworthy that all these plant hosts, including watermelon, tomato, cruciferous plants, flax, tulips, and melon, can be found cocultivated in the same geographical areas within oases (El-Saied et al., 2015; Mahmoud et al., 2022; Houssni et al., 2023). This adds an interesting dimension to our findings regarding the shared ancestry and presence of specific SIX genes in FOA isolate. The interconnectedness of these plants within oasis agriculture provides opportunity for interaction and genetic exchange, leading to coevolutionary dynamics that could have influenced the genetic makeup of FOA isolates and their associated SIX genes. Considering the unique ecological and agricultural conditions of oases, further investigations are needed to explore the distribution of ff. spp. in oasis agrosystems to understand these observed genetic dynamics of SIX genes.

In summary, the chromosome-level genomic resource developed in this study represent a significant milestone towards achieving a comprehensive understanding of the pathogen’s biology and the genetic mechanisms involved in FOA-date palm interaction. These packages will serve as a valuable genomic toolbox, facilitating the development of efficient diagnostic tools and enabling the analysis of FOA genetic diversity, ultimately contributing to a deeper understanding of FOA’s future impact on date palm groves.

## Data availability statement

The complete data from the current study was submitted at NCBI under the BioProject ID PRJNA658960 and BioSample ID SAMN15893572. The assembled Foa 44 genome has been deposited at DDBJ/ENA/GenBank under the accession JACSDM000000000. Users can download and reuse the data for research purpose only with an acknowledgement to us and quoting this paper as reference to the data.

## Author contributions

SK: Writing – original draft, Conceptualization, Data curation, Formal Analysis, Funding acquisition, Investigation, Methodology, Project administration, Resources, Software, Supervision, Validation, Visualization, Writing – review & editing. AA: Data curation, Formal Analysis, Investigation, Methodology, Resources, Software, Writing – review & editing. FG: Data curation, Formal Analysis, Methodology, Software, Writing – review & editing. IM-k: Methodology, Resources, Writing – review & editing. RL: Methodology, Writing – review & editing. MF: Methodology, Writing – review & editing, Data curation, Formal Analysis, Investigation. RM: Conceptualization, Formal Analysis, Funding acquisition, Investigation, Methodology, Project administration, Resources, Software, Supervision, Validation, Visualization, Writing – original draft, Writing – review & editing.
