# Supplementary figures and images for: Chromosome-scale assembly uncovers genomic compartmentation of Fusarium oxysporum f. sp. albedinis, the causal agent of Bayoud disease in date palm
Source: Front Microbiol. 2023 Oct 11;14:1268051. doi: 10.3389/fmicb.2023.1268051 (PMC10599148; doi:10.3389/fmicb.2023.1268051)

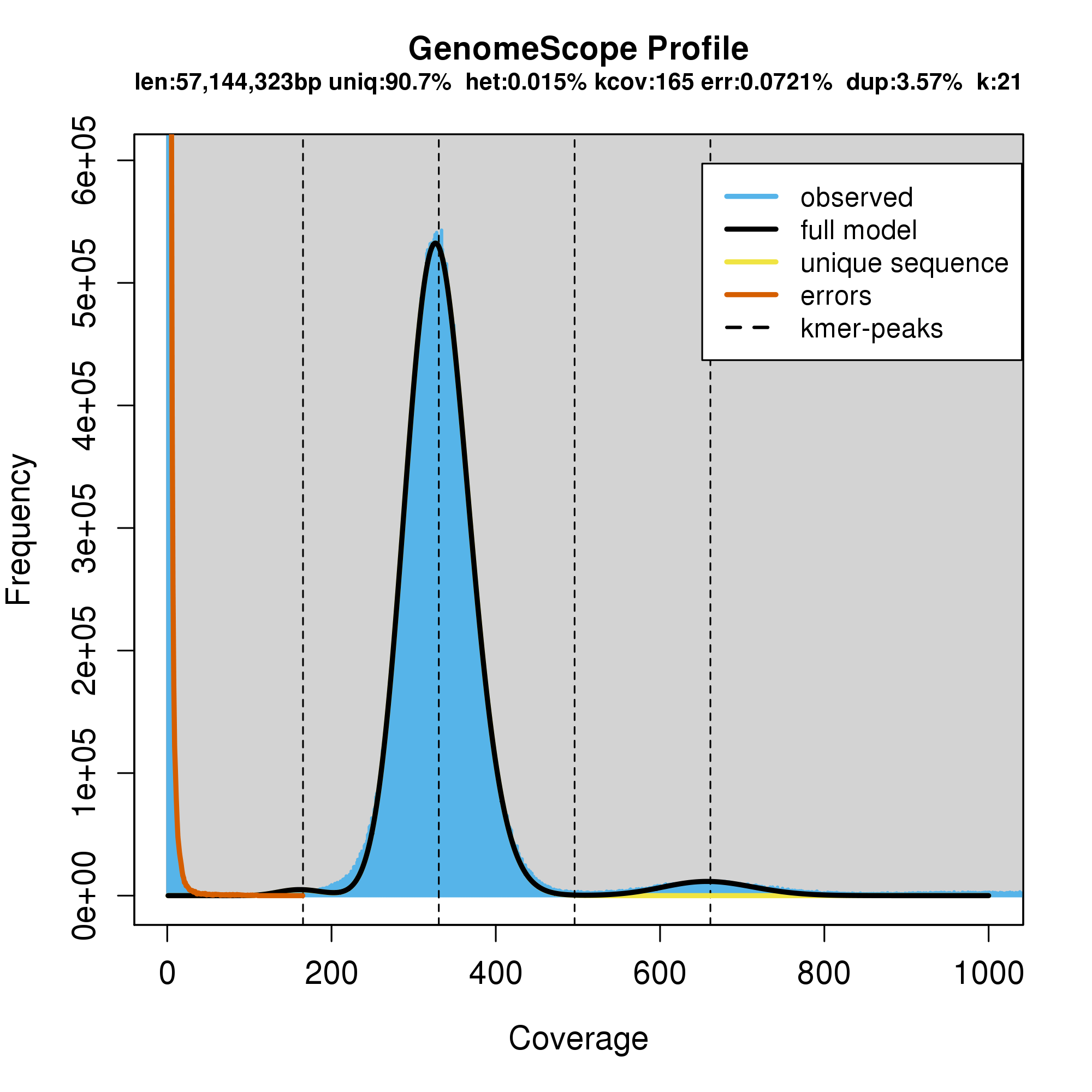

Supplement: Supplementary file 7 [file Image_1.PNG]

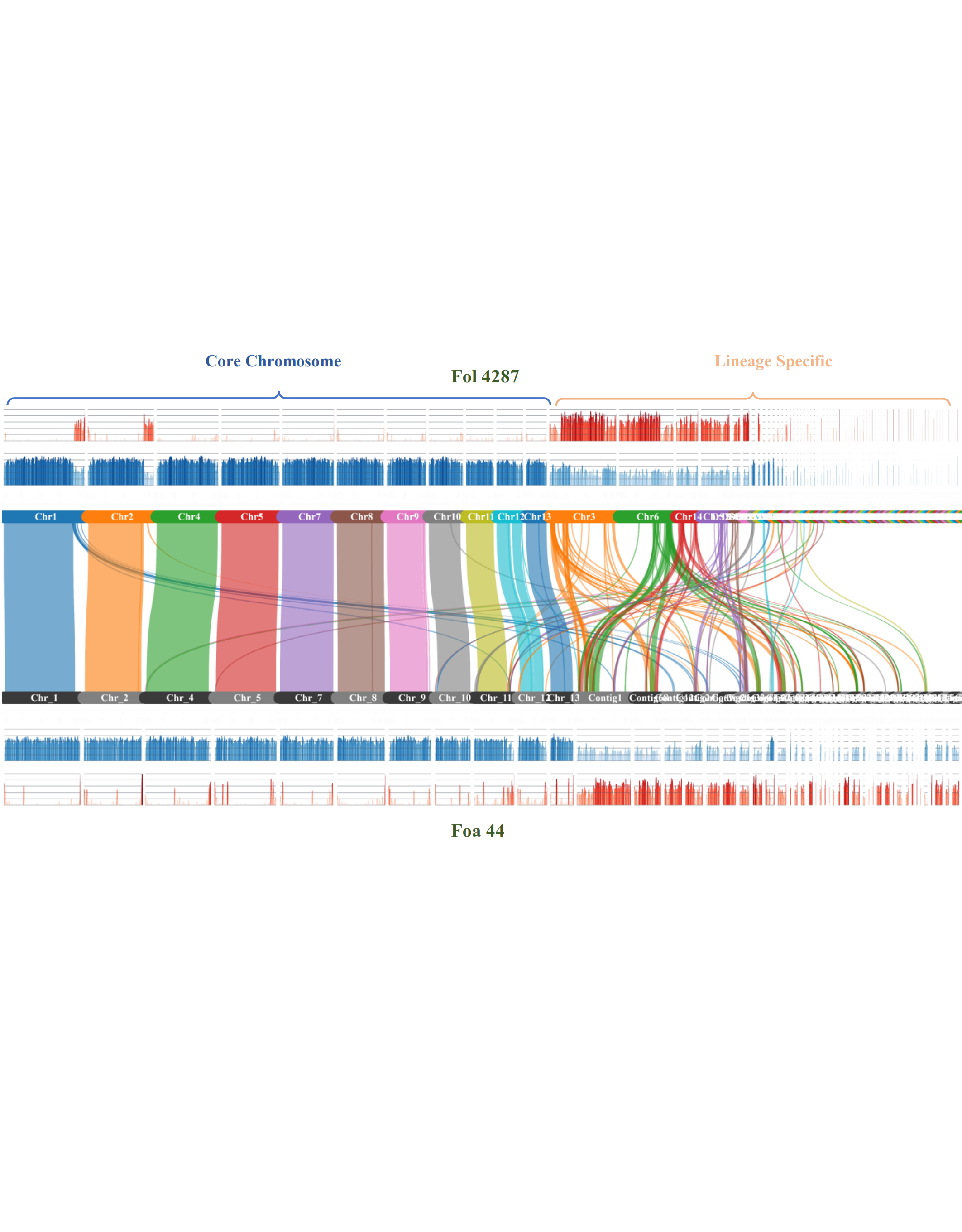

Supplement: Supplementary file 8 [file Image_2.TIF]

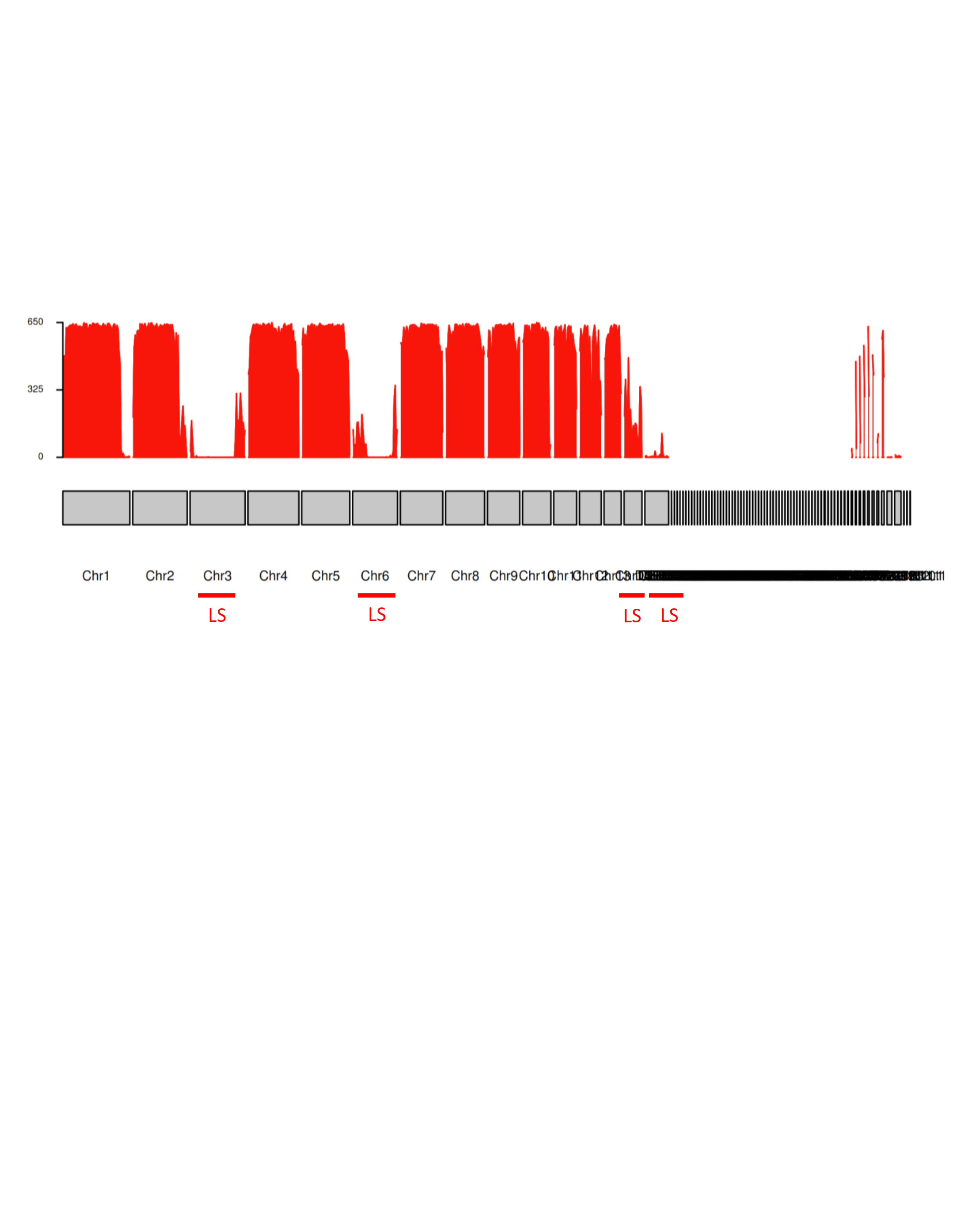

Supplement: Supplementary file 9 [file Image_3.TIF]
